# Using Digital Measurement–Based Care for the Treatment of Anxiety and Depression in Children and Adolescents: Observational Retrospective Analysis of Bend Health Data

**DOI:** 10.2196/46154

**Published:** 2023-04-20

**Authors:** Landry Goodgame Huffman, Darian Lawrence-Sidebottom, Jennifer Huberty, Monika Roots, Kurt Roots, Amit Parikh, Rachael Guerra, Jaclyn Weiser

**Affiliations:** 1 Bend Health Inc Beaverton, OR United States; 2 FitMinded Inc LLC Phoenix, AZ United States

**Keywords:** digital mental health intervention, anxiety, depression, child, adolescent, collaborative care, mental health, caregiver, pediatric, youth, demographic, health outcome, retrospective, treatment, e-mental health, symptoms

## Abstract

**Background:**

A growing body of evidence supports the efficacy of measurement-based care (MBC) for children and adolescents experiencing mental health concerns, particularly anxiety and depression. In recent years, MBC has increasingly transitioned to web-based spaces in the form of digital mental health interventions (DMHIs), which render high-quality mental health care more accessible nationwide. Although extant research is promising, the emergence of MBC DMHIs means that much is unknown regarding their effectiveness as a treatment for anxiety and depression, particularly among children and adolescents.

**Objective:**

This study uses preliminary data from children and adolescents participating in an MBC DMHI administered by Bend Health Inc, a mental health care provider that uses a collaborative care model to assess changes in anxiety and depressive symptoms during participation in the MBC DMHI.

**Methods:**

Caregivers of children and adolescents participating in Bend Health Inc for anxiety or depressive symptoms reported measures of their children’s symptoms every 30 days throughout the duration of participation in Bend Health Inc. Data from 114 children (age 6-12 years) and adolescents (age 13-17 years) were used for the analyses (anxiety symptom group: n=98, depressive symptom group: n=61).

**Results:**

Among children and adolescents participating in care with Bend Health Inc, 73% (72/98) exhibited improvements in anxiety symptoms and 73% (44/61) exhibited improvement in depressive symptoms, as indicated by either a decrease in symptom severity or screening out of completing the complete assessment. Among those with complete assessment data, group-level anxiety symptom T-scores exhibited a moderate decrease of 4.69 points (*P*=.002) from the first to the last assessment. However, members’ depressive symptom T-scores remained largely stable throughout their involvement.

**Conclusions:**

As increasing numbers of young people and families seek DMHIs over traditional mental health treatments due to their accessibility and affordability, this study offers promising early evidence that youth anxiety symptoms decrease during involvement in an MBC DMHI such as Bend Health Inc. However, further analyses with enhanced longitudinal symptom measures are necessary to determine whether depressive symptoms show similar improvements among those involved in Bend Health Inc.

## Introduction

In 2021, 5.6 million children and adolescents aged 3-17 years were diagnosed with anxiety and 2.4 million were diagnosed with depression. Although rates of anxiety and depression have increased steadily since 2016, 1 in 5 young people still do not receive adequate mental health care services [1]. As such, the demand for high quality and accessible mental health care for youth is more pressing than ever. In recent years, this demand has been addressed in part by the increased availability of mental health care via digital and telehealth platforms [2,3]. Several studies and meta-analyses have shown that digital mental health interventions (DMHIs) are as efficacious as in-person psychotherapy for the treatment of anxiety and depression [4-7], particularly among young people. Children and adolescents are accessing the internet at increasing rates and at increasingly younger ages [8]. Indeed, there is promising evidence that young people may glean more therapeutic benefits from DMHIs than their older counterparts due to their familiarity and comfort with web-based spaces as digital natives [9,10]. For example, in a systematic overview of 18 meta-analyses, Lehtimaki et al [4] found that computerized cognitive behavioral therapy interventions significantly improved mental and behavioral health problems in youth compared to those in nontreatment controls, with the most pronounced therapeutic effects among patients exhibiting anxiety and depressive symptoms.

Despite the promising evidence offered by burgeoning literature, the efficacy of DMHIs is still limited in several ways. These limitations largely fall within 2 categories: (1) lack of ability to meet clients’ and patients’ needs and (2) limited high quality evidence demonstrating the effectiveness of DMHIs. First, many DMHIs are administered using a standardized approach (eg, a single user interface or a treatment plan that does not adapt based on user responses), thus showing restricted ability to tailor their services to patients’ individual needs and circumstances [5,11]. This lack of adaptability restricts many DMHIs in their ability to address acute crises and issues of comorbidity [11]. Moreover, DMHIs are limited by their lack of human interaction. Hollis et al [12] and Grist et al [13] found that the involvement of a therapist or caregiver in the patient’s treatment plan significantly increases the therapeutic effects. Similarly, DMHIs that include supervision, such as those delivered in the context of a hospital, school, or therapy group, are far more likely to achieve clinically significant results [14]. The majority of DMHIs do not include human interaction, and thus, low patient engagement, high rates of dropout, and negligible improvements in symptoms remain the pressing issues [15,16]. Second, the evidence for DMHI’s effectiveness remains unclear and inconclusive largely due to the widespread lack of evidence-based practice utilized by DMHIs [13,17,18]. Although a number of DMHIs have been shown to effectively reduce youth anxiety and depression [4], methodological limitations such as sample size and quality of measures undermine the robustness of these findings [19]. This dearth of research also means that many questions remain regarding the basic factors associated with treatment effectiveness, such as the length of treatment and demographic and socioeconomic status [17]. This lack of methodological rigor paired with the issues of personalization and supervision mentioned above highlights the need for DMHIs to be both personalized and measurement based. These issues may be ameliorated by administering DMHIs within the context of collaborative care.

In recent years, collaborative care models have emerged as an effective framework for mental health care, particularly for the treatment of anxiety and depression [20-22]. The simplest collaborative care models are defined by a clear partnership between primary care providers (PCPs) and care teams, which comprise external behavioral health professionals (eg, case managers, therapists, coaches, psychiatrists) in order to facilitate comprehensive mental health treatment for an individual [23]. The additional support of the care team allows the PCP to delegate tasks to other team members while still tracking patient progress and optimizing treatment in real time. This optimization occurs via measurement-based care (MBC), which is another core component of the collaborative care model. MBC involves the frequent evaluation of patient symptoms and mental health status to allow for the continual tracking of patient progress. Additionally, MBC facilitates the prompt identification of treatment issues so that the DMHI can be adapted to address the patient’s existing needs.

Among adults, MBC consistently confers greater treatment effects than the traditional non-MBC across multiple types of diagnoses [24-26]. Although studies of MBC among children and adolescents remain scarce, they too offer promising results. In a 2017 meta-analysis of 12 studies [27], youth who were engaged in measurement-based mental health care tended to show greater improvements in symptoms than those treated using more traditional methods. Despite its clear utility as an effective tool for mental health treatment, both collaborative care models and MBC have been infrequently used within DMHIs. As such, little is known regarding the effectiveness of DMHIs in treating anxiety and depression among young people, particularly within the context of collaborative care and MBC.

The purpose of our study was to utilize member (eg, children, adolescents) data from a novel digital mental health company (Bend Health Inc) that administers MBC via collaborative care to determine the effects of an MBC DMHI on anxiety and depressive symptoms in children and adolescents. We hypothesized that both anxiety and depressive symptoms would decrease significantly over time of involvement with Bend Health Inc.

## Methods

### Study Design and Participants

Children (age 6-12 years) and adolescents (age 13-17 years) receiving treatment from Bend Health Inc, an MBC DMHI, between May 2022 and December 2022 were eligible for inclusion in this study. Scores on the Patient-Reported Outcomes Measurement Information System (PROMIS) validated anxiety and depression measures were used to determine whether a member should be included in the retrospective analysis. Specifically, members with baseline PROMIS scores indicating at least mildly severe symptoms of anxiety were included in the “elevated anxiety symptom severity” group, and members with baseline PROMIS scores indicating at least mildly severe symptoms of depression were included in the “elevated depressive symptom severity” group. Scoring and cutoff scores were determined by previously validated PROMIS scoring norms [28,29]. Many members included in our analyses exhibited additional comorbidities, including attention-deficit/hyperactivity disorder, mania, and posttraumatic stress disorder. The rates of comorbidities are reported in Multimedia Appendix 1.

### Ethical Considerations

All Bend Health, Inc. members above the age of 12 (adolescent members and participating caregivers) complete informed consent prior to enrolling in services. Caregivers consent on behalf of their children ages 12 and under. The informed consent process includes essential information about Bend Health, Inc.’s telemedicine services and privacy policies. Given the current study was a retrospective analysis, it was classified as exempt from consent requirements under human subjects review and approved by BRANY IRB (Study ID 23-12-034-1374, 16 January 2023). Study data were de-identified and stored on a HIPAA–compliant online drive using industry standard encryption. Participants received no additional compensation for participation.

### Treatment

Bend Health Inc is an MBC DMHI for children and adolescents (age 2-17 years) based on a measurement-based collaborative care model. Most current Bend Health Inc members enroll through a pediatric PCP referral. PCPs remain closely involved in members’ care throughout their time at Bend Health Inc, helping to determine and execute the member’s care and receiving updates on the member’s progress up to 2 times per month (once after a monthly psychiatric provider session, if applicable, and once at the end of each month). Outside of PCP referrals, there are several other pathways to enrollment at Bend Health Inc: enrolling in employer benefits, enrolling in insurance benefits, and paying a monthly fee (direct to consumer).

Bend Health Inc uses a team-based treatment approach, leveraging regular involvement from PCPs, mental health professionals, and caregivers to holistically treat mental and behavioral health problems among youth members. After a member is enrolled and assessed, a behavioral care coordinator coordinates with their PCP as well as other relevant care team members (eg, psychiatrist, therapist, coach) to determine the member’s care program (eg, the plan of care developed based on a member’s presenting symptoms and age). The behavioral care coordinator then oversees the execution of the member’s care program under the direction of the PCP. Each member’s care program includes synchronous video-based (virtual) care sessions between the member and a coach or therapist, asynchronous instant messaging with their coach or therapist, and access to informational resources via the web-based platform. In general, care programs last between 4 and 6 months, as determined by the member’s care team. However, members can transition to a new care program if their ongoing symptoms indicate that they would be more benefitted by another program; as such, a member may begin with a depression-oriented care program and transition to an attention-deficit/hyperactivity disorder–oriented care program depending on their symptom presentation.

Care programs are adapted to be developmentally appropriate for those receiving care from Bend Health Inc, with modifications in the care program based on member age. For example, programs for children (members aged 12 years or younger) require an adult caregiver to be present in synchronous sessions, during which the caregiver actively engages with and assists their child throughout the program. Programs for adolescents (members aged 13-17 years) do not require caregivers to attend sessions with their child. However, adolescent programs still include aspects that involve and support the caregiver during and between sessions, and caregivers are still required to be readily accessible throughout sessions (eg, in the same general area). Program components such as scripts and tasks are also adapted to match cognitive and emotional abilities across a range of ages, such that a child-oriented program includes simpler language and tasks (eg, drawing vs writing) and an adolescent-oriented program includes more complex language and tasks.

All members may participate in up to 5 sessions per month with any of the following health care providers (depending on treatment plan and insurance coverage): behavioral care coordinator, coach, therapist, or psychiatrist. Coaching sessions are 30 minutes in duration, and members may attend a total of up to 2-3 coaching or therapy sessions per month as part of their 5 sessions allowed per month. Caregivers of Bend Health Inc members have access to web-based one-on-one asynchronous messaging with the behavioral care coordinator, coach, therapist, or psychiatrist. Every 30 days, caregivers are asked to complete web-based assessments of mental health outcomes, including symptoms of depression and anxiety. Due to variations in caregivers’ responsivity and availability to complete assessments, the rates of interassessment duration vary.

Bend Health Inc coaching sessions are intended to provide the members and their families with appropriate evidence-based behavior change tools, help members with self-reflection, strengthen self-efficacy and autonomy, and, when appropriate, serve as a gateway to additional mental health support via sessions with a licensed therapist. Therapy sessions are intended to provide diagnostic clarity to inform a clinical framework, uncover potential sources of unwanted and targeted behaviors, and address trauma or other complicated clinical psychopathology. Coaching and therapy sessions are based upon cognitive behavioral therapy, behavioral activation, parent management training, mindfulness-based cognitive therapy, motivational interviewing, and mindfulness-based stress reduction. All Bend Health Inc coaches and therapists are trained in these modalities.

### Study Measures

Upon enrollment, caregivers are asked to complete screening questions for depression, anxiety, and other mental and behavioral health symptoms. Anxiety and depression screener questions were drawn from the Diagnostic and Statistical Manual of Mental Disorders, fifth edition, text revision Cross-Cutting Symptom Measure for children aged 6-17 years [30]. These screeners are intended to flag members with depressive or anxiety symptoms while minimizing the workload for caregivers of members less likely to have these symptoms. For anxiety symptoms*,* the screening question items are as follows: [my child has] (1) said they felt nervous, anxious, or scared, (2) not been able to stop worrying, and (3) said they could not do things they wanted to or should have done because it made them feel nervous. For depressive symptoms, the screening question items are as follows: [my child has] (1) had less fun doing things than they used to and (2) seemed sad or depressed for several hours. If the response to any of the screening questions is “almost never” or more (eg, a raw value of 2 or greater), caregivers are required to complete the entire depression or anxiety PROMIS measure. The depression and anxiety PROMIS measures were developed for caregivers of children aged 6-17 years [28,29]. The PROMIS depression measure has 11 questions, and the anxiety measure has 10 questions. After being prompted with, “during the past 2 weeks, how much (or how often) has your child,” caregivers select the best-fit response to each item using a 5-item Likert scale (ranging from “not at all” to “nearly every day”). Assessment scores are reported to the caregiver and care team members on a web-based member portal and used to guide the patient’s care plan. To increase the accuracy of symptom reports, caregivers are explicitly instructed to complete screening questions and assessments with their child alongside them (“Be sure you have your child or teen with you. You’ll be answering a series of questions that will be used to create your personalized care plan.”). Additionally, at enrollment, caregivers were asked to report their child or adolescent’s demographic information, including age, sex at birth (male or female), and race/ethnicity (American Indian or Alaska Native, Asian, Black or African American, Hispanic or Latino, Native Hawaiian or other Pacific Islander, White, or Other).

### Statistical Analysis

The total raw scores for the depression and anxiety PROMIS assessments were calculated by adding the individual scores of all items. Raw scores were converted to standardized T-scores based on established criteria [28,29]. For both questionnaires, T-scores less than 55 indicated a nonclinically significant level of depression or anxiety (no to slight symptom severity), T-scores between 55 and 59.9 indicated mild symptom severity, T-scores between 60 and 69.9 indicated moderate symptom severity, and T-scores exceeding 70 indicated severe symptoms. In all descriptions of the symptom severity classifications, scores of those who were screened out of completing the full assessment(s) (due to no longer reporting anxiety or depressive symptoms per screener questions) were classified as screened out. Because of this limitation, 2-tailed *t* tests were used to determine whether those who screened out at later assessments showed lower symptom severity at baseline.

Each member’s δ (change) score was calculated as the final T-score (last assessment) – baseline T-score (first assessment) to quantify the change in the T-score from baseline to the end of treatment. Negative change scores indicated an improvement (decrease) in symptom severity. One-tailed Wilcoxon signed-rank tests were conducted on complete assessment data to determine whether change scores for depression and anxiety were significantly less than 0. For other descriptive statistics of change in symptom severity, members were considered to show improvement if they either (1) exhibited a decrease in anxiety or depressive scores or (2) were screened out of completing an entire assessment based on reports of low symptom severity in the screener questions.

Changes in depression and anxiety T-scores over time were further assessed by linear mixed-effects models with a fixed effect of time of assessment (eg, days from baseline) and a random effect of member (identification number) on the intercept. The number of days from baseline was used as the time variable because there was variability in the interassessment duration (eg, some members’ second assessments took place 25 days after baseline while others’ assessments took place 40 days after baseline). The average number of treatment sessions per month (calculated as time between the first and last assessments divided by the number of sessions with a coach, therapist, or psychiatrist) was added to this basic model as an additional predictor, and the alternative model was compared to the basic model by a likelihood ratio test. If the likelihood ratio test indicated that the predictor improved model fit (eg, *P*<.05), it was retained in the final model. Finally, analysis of variance was performed on the model effects to determine whether each effect was significant. The primary linear mixed-effects models for depressive and anxiety symptoms were first performed on only complete assessment data for all members. Then, follow-up linear mixed-effects models were conducted on those with complete data for the first 3 assessments (eg, completed assessments at baseline, assessment 2, assessment 3). These follow-up sensitivity analyses were performed to test the robustness of findings to dropout effects and are reported in Multimedia Appendix 1.

For all analyses, group trends were reported with standard descriptive statistics, including percent of sample (%), mean (SD), and median (IQR). Due to low response rates in the anxiety measure for assessments 5-7 for the elevated anxiety group, descriptive statistics for anxiety T-scores are not reported past assessment 4. Similarly, due to the low response rates in the depression measure for assessments 4-5 for the elevated depressive symptom group, descriptive statistics for depression T-scores are not reported past assessment 3.

## Results

### Baseline Characteristics of the Participants

A total of 114 members (age 6-17 years) met the inclusion criteria for elevated symptoms of anxiety or depression, with 85.9% (98/114) included in the anxiety analyses and 53.5% (61/114) included in the depression analyses. Approximately 42.1% (48/114) of all the members in this study met the inclusion criteria for both analyses. Due to variations in the rates of completion of screeners versus assessments as well as the total duration of participation with the DMHI, the rates of PROMIS measure completion decreased over assessments for both symptom groups. All included members completed the first assessment (baseline), and few members completed the subsequent assessments (see Table 1).

The members in the group with elevated anxiety symptoms were 11.9 (SD 3.3) years old, with adolescents comprising approximately half of the group (51/98, 52%). Approximately two-thirds of the anxiety group were females (66/98, 67%). Most members in the anxiety group identified as White (57/98, 58%) or other (31/98, 32%). The members in the group with elevated depressive symptoms were 13.2 (SD 2.7) years old, with a larger proportion of adolescents than children (45/61, 74%). The depression group consisted of predominantly females (43/61, 71%). Most members in the depression group identified their race/ethnicity as White (33/61, 54%) or other (22/61, 36%). Table 2 shows the comprehensive demographic information of the anxiety and depressive symptom groups at baseline.

**Table 1:** Rates of Patient-Reported Outcomes Measurement Information System measure completion for depression and anxiety groups.

Assessment number	Elevated depressive symptom group (n=61), n (%)		Elevated anxiety symptom group (n=98), n (%)	
	Complete assessment	Screener or complete assessment	Complete assessment	Screener or complete assessment
1 (baseline)	61 (100)	61 (100)	98 (100)	98 (100)
2	12 (20)	25 (41)	34 (35)	44 (45)
3	3 (5)	13 (21)	21 (21)	22 (22)
4	1 (2)	5 (8)	7 (7)	9 (9)
5	1 (2)	3 (5)	2 (2)	3 (3)
6	0 (0)	2 (3)	0 (0)	1 (1)

**Table 2:** Demographic information of the members in the depression and anxiety groups at baseline.

Demographics		Elevated depressive symptom group (n=61)	Elevated anxiety symptom group (n=98)
**Age (years), mean (SD)**		13.2 (2.7)	11.9 (3.3)
	Child (6-12 years), n (%)	16 (26)	47 (48)
	Adolescent (13+ years), n (%)	45 (74)	51 (52)
**Sex, n (%)**			
	Female	43 (71)	66 (67)
	Male	18 (30)	31 (32)
**Race/ethnicity, n (%)**			
	White	33 (54)	57 (58)
	Other	22 (36)	31 (32)
	Asian	5 (8)	8 (8)
	Hispanic/Latino	0 (0)	1 (1)
	Black/African American	1 (2)	1 (1)
	American Indian or Alaska Native	0 (0)	0 (0)

### Elevated Anxiety Symptom Severity Group

While receiving care from Bend Health Inc, members in the elevated anxiety symptom group attended 0-19 (median 0 [IQR 2]) sessions with a coach, therapist, or psychiatrist. Those in the elevated anxiety symptom group who completed at least 2 assessments (eg, screeners or complete measures; n=45) attended a median of 2 (IQR 3) sessions with a coach, therapist, or psychiatrist and an average of 1.50 (SD 0.73; range 0-3.6) sessions per month. Members completed their first and last assessments between 0 and 221 days apart (median 0 [IQR 1.5] sessions). The duration between the assessments ranged from 8 to 90 days, with the central tendency approximately equal to a month (median 34 [IQR 15] days).

**Figure 1:**
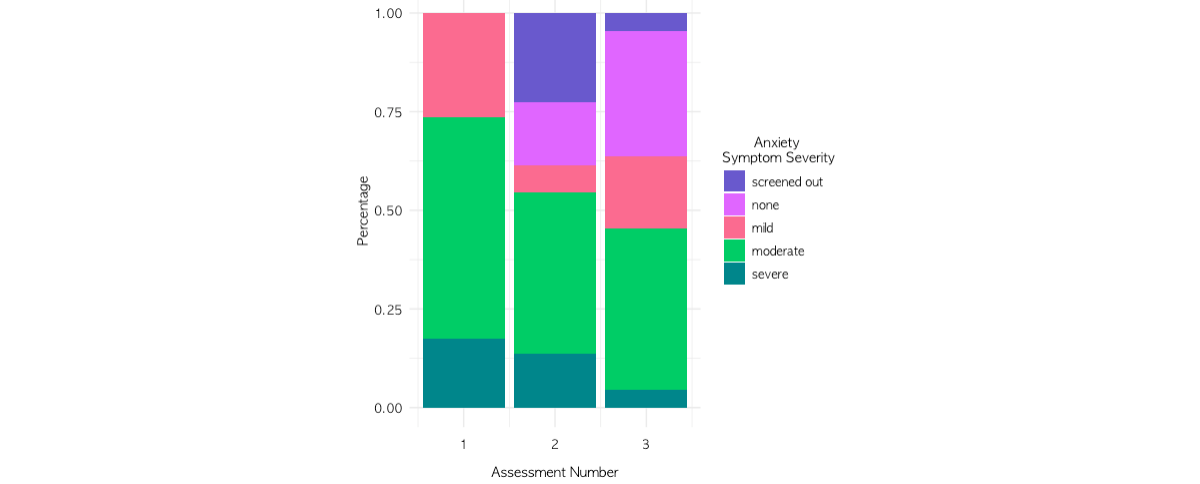
Distribution of anxiety symptom severity categories across assessments (including those who screened out of the assessment, indicated in purple).

Members with elevated anxiety symptoms had a mean PROMIS anxiety T-score of 64.97 (SD 6.71) at baseline, which is classified as moderate anxiety symptom severity. For members who completed at least 2 screeners or complete assessments, 73% (72/98) had improvements in anxiety symptom severity from baseline to their last assessment, as indicated by either a decrease in symptom severity scores or screening out of completing the entire assessment. Indeed, rates of moderate to severe anxiety symptom severity decreased across assessments, and rates of screening out of the complete assessment increased across assessments (Figure 1 and Table 3). Specifically, 23% (10/44) of the members with elevated anxiety symptoms at baseline screened out of the complete second anxiety PROMIS assessment, and 5% (1/22) screened out of completing the third anxiety PROMIS assessment. PROMIS anxiety T-scores at baseline for participants who screened out of the second PROMIS assessment (mean 61.78 [SD 7.14]) were slightly lower than those of participants who took the complete assessment (mean 65.89 [SD 5.82]), with the *t* test trending toward statistical significance (*t*_44_=1.88; *P=*.07). For those with at least one complete follow-up assessment (n=38), anxiety symptoms decreased from the first (mean 65.97 [SD 6.27]) to the last assessment (mean 61.28 [SD 9.92]; *t*_37_*=–*3.12; *P=*.002).

**Figure 2:**
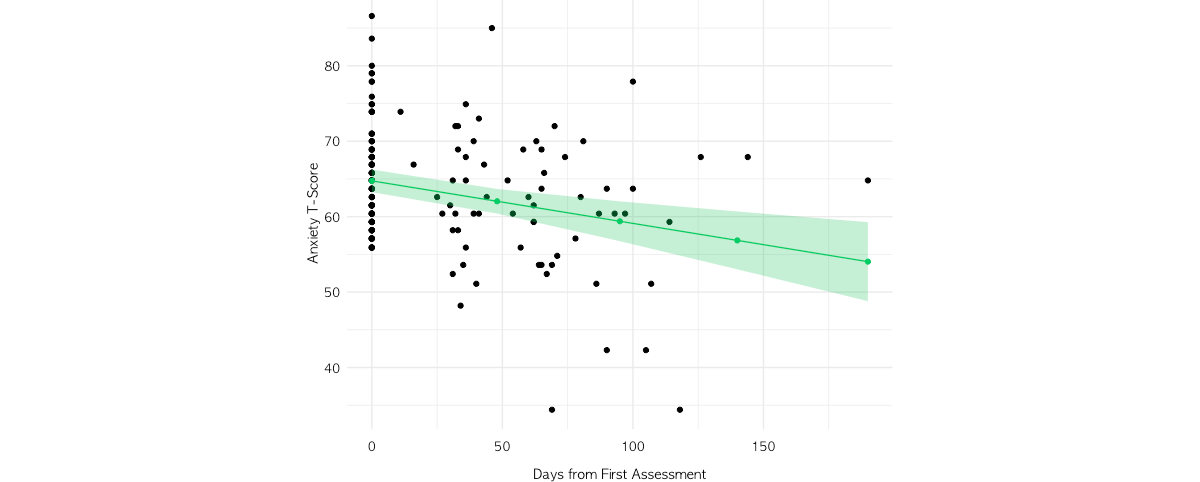
Linear mixed-effects model results demonstrating the main effect of days from first assessment on anxiety T-scores.

In the linear mixed-effects model of those with complete PROMIS anxiety assessment data (eg, excluding those at each assessment who had completed the screener only), the addition of number of sessions per month (with a coach, therapist, or psychiatrist) as a predictor did not improve model fit (*χ*^2^_1_=1.6; *P=*.20). Thus, the final model included the main time variable (days from baseline) as a fixed effect and member as a random effect on the intercept (Figure 2, Table 4). The model explained 10.4% of the total variance, and the main effect of days from the baseline was statistically significant (*F*_1,64_=14.25; *P<*.001; Figure 2). Specifically, the model estimated a decrease in anxiety T-score of 0.06 points per day (1.8 points per month). When the linear mixed-effects analysis was repeated on assessments 1 through 3 for those with at least 3 complete assessments (n=17), the results did not differ substantively from the primary model results (Multimedia Appendix 1).

**Table 3:** Rates of Patient-Reported Outcomes Measurement Information System anxiety symptom severity categories across assessments.

Anxiety symptom severity	Assessment 1 (n=98), n (%)	Assessment 2 (n=44), n (%)	Assessment 3 (n=22), n (%)
Screened out (no complete assessment)	0 (0)	10 (23)	1 (5)
None	0 (0)	7 (16)	7 (32)
Mild	26 (27)	3 (7)	4 (18)
Moderate	55 (56)	18 (41)	9 (41)
Severe	17 (17)	6 (14)	1 (5)

**Table 4:** Results of the linear mixed-effects model for the anxiety symptom group.^a^

Predictors			Anxiety T-score estimates		*P* value
(Intercept) (95% CI)			64.74 (63.24 to 66.25)		<.001^b^
Days from baseline, estimate (95% CI)			–0.06 (–0.09 to –0.03)		<.001^b^
**Random effects**					
	σ^2^	37.64		N/A^c^	
	τ_00_^d^	22.49		N/A	

^a^Total sample size at baseline=98; 163 observations; marginal *R*^2^=0.104.

^b^Statistically significant effects (*P<*.05).

^c^N/A: not applicable.

^d^Random intercept variance (also known as between-individual variance).

### Elevated Depressive Symptom Severity Group

While receiving care from Bend Health Inc, members in the elevated depressive symptom group attended 0-10 (median 0 [IQR 2]) sessions with a coach, therapist, or psychiatrist. Those in the elevated depression symptom group who completed at least 2 screeners or full assessments (n=26) attended a median of 3 (IQR 2.75) sessions with a coach, therapist, or psychiatrist with an average of 1.45 (SD 0.68; range 0-2.6) sessions per month. Members completed their first and last screeners or complete assessments between 0 and 168 days apart (median 0 [IQR 62] days). The duration between screeners only or complete assessments ranged between 20 and 90 days, with the central tendency approximately equal to a month (median 32 [IQR 17] days).

**Figure 3:**
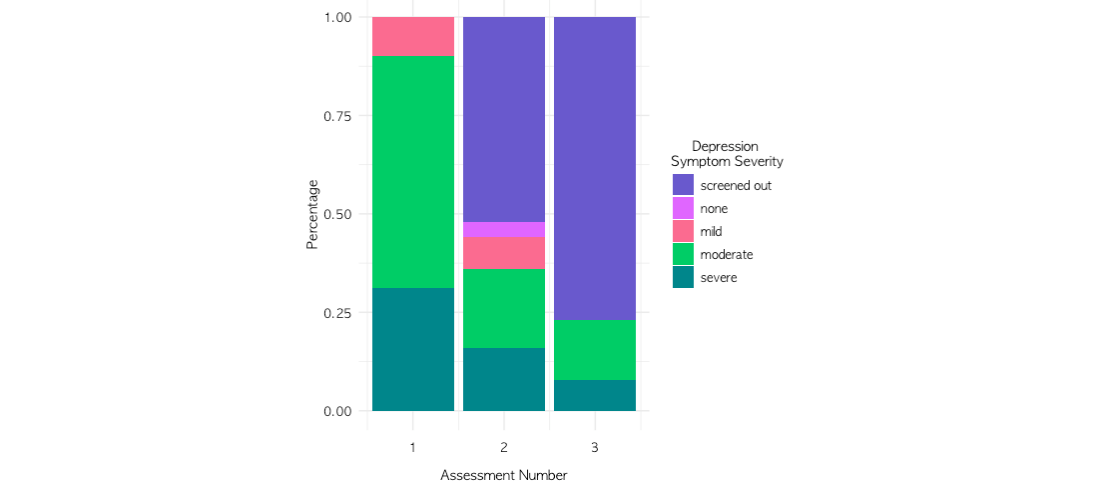
Distribution of depressive symptom severity categories across assessments (including those who screened out of the assessment, indicated in purple).

Members with elevated depressive symptoms had a mean PROMIS depression T-score of 68.20 (SD 7.48) at baseline, which is classified as moderate depressive symptom severity. For members who completed at least 2 screeners or complete assessments, 73% (44/61) had improvements in depressive symptom severity from baseline to their last assessment, as indicated by either a decrease in symptom severity scores or screening out of completing the complete assessment. Indeed, the rates of more severe depressive symptoms decreased across assessments and the rates of screening out of the complete assessment increased across assessments (see Figure 3 and Table 5). Specifically, 52% (13/25) of the members with elevated depressive symptoms at baseline screened out of the complete second depression PROMIS assessment, and 77% (10/13) screened out of completing the third depression PROMIS assessment. Although PROMIS depression T-scores were slightly lower for those who screened out of the second PROMIS assessment (mean 66.08 [SD 4.42]) versus those who took the complete assessment (mean 68.00 [SD 8.27]), this difference was not significant (*t*_24_=0.97; *P=*.34). For those with at least one complete follow-up assessment (n=13), depressive symptoms remained stable at moderately severe from the first (mean 68.20 [SD 8.33]) to the last assessment (mean 68.02 [SD 9.35]; *t*_12_*=–*0.08; *P=*.47).

**Table 5:** Rates of Patient-Reported Outcomes Measurement Information System depressive symptom severity categories across assessments.

Depressive symptom severity	Assessment 1 (n=61), n (%)	Assessment 2 (n=25), n (%)	Assessment 3 (n=13), n (%)
Screened out (no complete assessment)	0 (0)	13 (52)	10 (77)
None	0 (0)	1 (4)	0 (0)
Mild	6 (10)	2 (8)	0 (0)
Moderate	36 (59)	5 (20)	2 (15)
Severe	19 (31)	4 (16)	1 (8)

**Figure 4:**
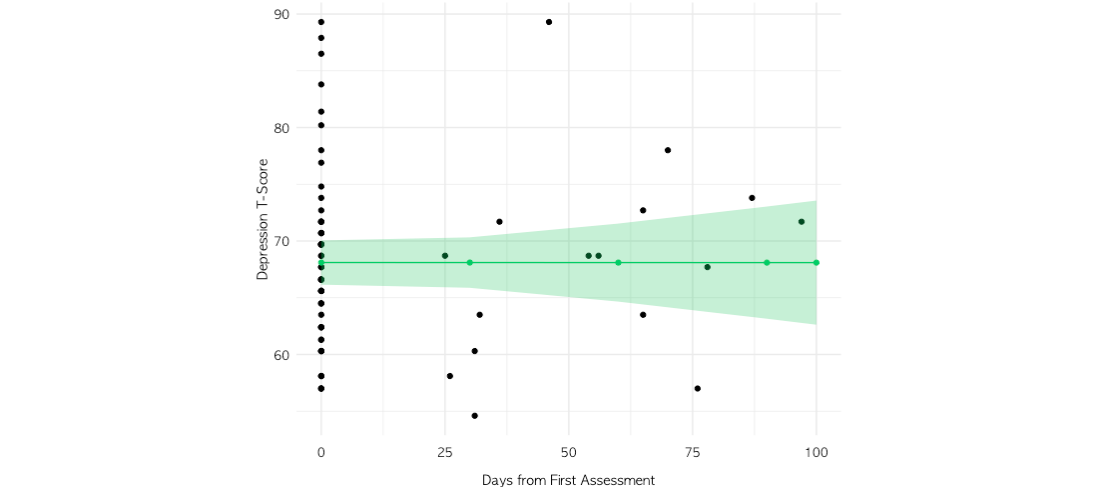
Linear mixed-effects model results demonstrating the main effect of days from first assessment on depression T-scores.

In the linear mixed-effects model of complete PROMIS depression assessment data (excluding those at each assessment who had completed the screener only), the addition of the number of sessions per month (with a coach, therapist, or psychiatrist) as a predictor did not improve model fit (*χ*^2^_1_=2.6; *P=*.11). Thus, the final model included the main time variable (days from baseline) as a fixed effect and member as a random effect on the intercept (Figure 4, Table 6). The model explained none of the total variance (0%), and the main effect of days from the baseline was not significant (*F*_1,16_=1.02, *P*>.99; Figure 2). When the linear mixed-effects analysis was repeated on assessments 1 through 3 for those with at least 3 complete assessments, the results did not differ substantively from the primary model results (Multimedia Appendix 1).

**Table 6:** Results of the linear mixed-effects model for the depressive symptom group.^a^

Predictors		Depression T-score estimates	*P* value
(Intercept) (95% CI)		68.10 (66.15 to 70.06)	<.001^b^
Days from baseline, estimate (95% CI)		0.00 (–0.06 to 0.06)	.99
**Random effects**			
	σ^2^	32.59	N/A^c^
	τ_00_^d^	26.92	N/A

^a^Total sample size at baseline=61; 78 observations; marginal *R*^2^=0.000.

^b^Statistically significant effects (*P<*.05).

^c^N/A: not applicable.

^d^Random intercept variance (also known as between-individual variance).

## Discussion

### Principal Results

The purpose of this study was to utilize member data from Bend Health Inc to determine the effects of an MBC DMHI on depressive and anxiety symptoms among children and adolescents. To our knowledge, this is the first study evaluating the effects of a DMHI that uses a measurement-based collaborative care model to reduce symptoms of depression and anxiety in children and adolescents over time. We found that youth receiving care from Bend Health Inc exhibited improvements in both anxiety and depressive symptoms over time. Notably, depressive symptoms remained largely stable over time when assessing only those who did not screen out (eg, those with higher depressive symptoms at later assessments). As such, more comprehensive measures are necessary to fully elucidate the effects of Bend Health Inc on those with more severe depressive symptoms. Given that depressive symptoms and episodes often increase in early adolescence [31-33], it is notable that depression severity remained stable even among Bend Health Inc members with persistent symptoms.

Depressive and anxiety symptom frequency and severity decreased between baseline and subsequent assessments in child and adolescent members who were receiving care with Bend Health Inc. However, the method in which members’ symptoms were assessed introduced significant nuances into our study design and interpretation of results. Members exhibited improvements at subsequent assessments via one of the 2 pathways: by showing decreases in symptom scores or by screening out of symptom measures entirely. When considering the full cohort together, including those who completed the full measures and those who screened out, we observed, across both anxiety and depressive symptom groups, (1) decreased frequency of moderate and severe symptoms and (2) increased frequency of those whose symptoms were low enough to screen out of the full measures. Specifically, 73% (72/98) of the anxiety symptom cohort and 73% (44/61) of the depressive symptom cohort either showed a decrease in symptom severity or screened out between their first and final assessments. However, when confining our analyses to those with only completed assessments, only anxiety symptoms showed significant decreases over time. Given that completion of the full symptom assessments was dependent upon elevated severity of symptoms (as proxied by the screener questions), analyzing only those with completed assessments may have naturally highlighted those with the most persistent and severe symptoms. Moreover, the depressive symptom group was particularly limited by low power to detect changes over time, as over half of those who exhibited elevated depressive symptoms at baseline screened out by the second assessment. More comprehensive symptom measures are necessary to untangle the nuances of our findings.

Despite these limitations, our results are still promising. As an increasing number of young people and families seek DMHIs over traditional mental health treatments due to their accessibility and affordability, this study offers preliminary evidence that MBC DMHIs such as Bend Health Inc have the potential to mitigate anxiety and depressive symptoms in those younger than 18 years. Previous meta-analyses of DMHIs have found that interventions involving supervision, such as asynchronous video calls or follow-ups by telephone or instant messaging, are associated with greater improvements in depressive and anxiety symptoms when compared to unsupervised self-guided interventions [14,18]. Therefore, future research of MBC DMHIs ought to compare various care methodologies such as those involving both supervised and unsupervised care.

We found that the length of involvement in Bend Health Inc care was the foremost predictor of anxiety symptom severity over time, such that members’ anxiety symptoms decreased as their duration of participation increased. Several studies have investigated the complex associations between DMHI length and symptom improvement among youth, with some suggesting that longer involvement in therapy (eg, number of months or hours involved in intervention) is associated with larger reductions in symptoms [34,35]. Conversely, some studies have found that the positive effects of various care programs on symptom severity are the greatest among interventions of 1-2 months in duration [36] or are not related to treatment duration at all [12]. Our study supports the former finding that length of treatment is indeed closely linked to changes in symptom severity among Bend Health Inc members. Because the length of involvement in our sample was relatively brief, with the average duration of treatment equaling just longer than a month for those with anxiety or depressive symptoms, further studies on Bend Health Inc patients who have engaged in treatment for longer periods of time (eg, 4-6 months) are necessary to determine the full scope of the association between duration of time in treatment and symptom improvement.

### Limitations and Future Directions

Our study is limited by several factors. Primarily, our findings are limited by a lack of specificity in symptom measurement. To reduce member and caregiver burden, those who did not report significant anxiety or depression symptoms using preliminary screener questions were not given the opportunity to complete the full anxiety and depression questionnaires. As such, we did not have complete assessment data for most members who exhibited low anxiety and depressive symptoms after their first assessment. This lack of data among those arguably exhibiting the largest improvements likely skewed our longitudinal analyses to primarily reflect those with more persistent and severe symptoms. However, it should be noted that depressive symptoms often exhibit marked increases in early adolescence [31-33]. Conversely, Bend Health Inc members who continued to report elevated depressive symptoms exhibited stability in their symptoms over time. When compared to commonly observed increases among untreated adolescents, this stability offers promising evidence that Bend Health Inc programs are beneficial even for youth with persistent depressive symptoms. Moreover, our study is limited by its use of caregiver-reported assessments of child and adolescent symptoms. Although there is a precedent for using observer (eg, caregiver, clinician) ratings to track symptom progression in MBC [37-39], evidence also suggests that caregiver reports may not capture potential internalizing problems as effectively as youth self-report [40]. Follow-up studies that include both adolescent-reported and caregiver-reported metrics are necessary to determine whether our results are robust to reporters.

Many studies evaluating DMHI effectiveness have compared patients receiving treatment to nonactive controls (eg, those participating in no intervention). Subsequent studies of Bend Health Inc could be improved by the inclusion of various levels of treatment in order to test whether care with Bend Health Inc confers greater effects than alternatives (eg, no treatment, traditional face-to-face treatment, nonmeasurement-based DMHIs). Furthermore, the duration of care in our study was relatively moderate and highly variable, with average participation lasting approximately a month but ranging from 0 to 168 days. Although our follow-up analyses of members with 3 or more assessments suggest that decreases in symptoms are robust to dropout effects, the rigor of future studies can be improved by increasing the number of participants with longer involvement and more assessment points.

Importantly, our study did not have adequate power to investigate differences in symptom change across demographic groups such as age, gender, race, and ethnicity. A number of studies have demonstrated that the risk for anxiety and depression increases in adolescence [41]. Moreover, females often report higher rates of anxiety and depression than males, despite depression and suicide being one of the leading causes of death among men [42-44]. Mental health disparities between racial and ethnic groups have also been reported, although findings are inconsistent about which group is the most at risk [43,45]. Further studies are necessary to determine whether those involved in an MBC DMHI like Bend Health Inc exhibit similar demographic differences. Information generated by these future studies will be crucial to informing care programs at Bend Health Inc and other DMHIs.

The principal finding of this study indicates that children and adolescents involved in Bend Health Inc show a significant reduction of anxiety and depressive symptoms over time. As such, our study offers preliminary evidence suggesting that MBC DMHIs such as Bend Health Inc may aid in reducing anxiety and depressive symptoms in youth. Future studies bolstered by improved measurement of symptoms and a larger and more diverse cohort of youth are paramount in order to establish the effectiveness of Bend Health Inc as an evidence-based provider of DMHIs in the United States.

## Appendix

Multimedia Appendix 1 Supplementary data.

## Abbreviations

DMHI: digital mental health intervention
MBC: measurement-based care
PCP: primary care provider
PROMIS: Patient-Reported Outcomes Measurement Information System
