# Supplementary material for: Using Digital Measurement–Based Care for the Treatment of Anxiety and Depression in Children and Adolescents: Observational Retrospective Analysis of Bend Health Data
Source: JMIR Pediatr Parent. 2023 Apr 20;6:e46154. doi: 10.2196/46154 (PMC10160939; doi:10.2196/46154)
Supplement: Multimedia Appendix 1 [file pediatrics_v6i1e46154_app1.docx]

# Multimedia Appendix 1

## Comorbidities

Comorbidities are very common in the current sample. Among those exhibiting elevated depressive symptoms at baseline, 57 had at least one other elevated symptom at baseline, including anxiety (n=48), ADHD-Inattention (n=29), ADHD-Hyperactivity (n=14), Opposition/Defiance (n=24), mania (n=4), and PTSD (n=25).

Among those exhibiting elevated anxiety symptoms at baseline, 80 had at least one other elevated symptom at baseline, including depression (n=98), ADHD-Inattention (n=45), ADHD-Hyperactivity (n=22), Opposition/Defiance (n=32), mania (n=8), and PTSD (n=34).

## Secondary Analyses

To determine whether results of our linear mixed-effects models were robust to effects of dropout, we conducted secondary analyses that included only those with three or more assessments. For both anxiety and depressive symptoms, the results of these analyses were largely consistent with the primary analyses.

| Table S1. Results of linear mixed-effects model for anxiety symptom group with three or more assessments | | | |
| --- | --- | --- | --- |
|  | Anxiety T-score | | |
| *Predictors* | *Estimates* | *CI* | *P* |
| (Intercept) | 65.80 | 62.34 - 69.27 | **<.001** |
| Days from baseline | -0.09 | -0.13 - -0.04 | **<.001** |
| Random effects |  |  |  |
| σ^2^ | 26.99 |  |  |
| τ_00_ | 28.90 |  |  |
| N _Member ID_ | 17 |  |  |
| Observations | 51 |  |  |
| Marginal R^2^ / Conditional R^2^ | 0.228 / NA |  |  |

*Note.* Statistically significant effects (*P* < .05) are bolded.


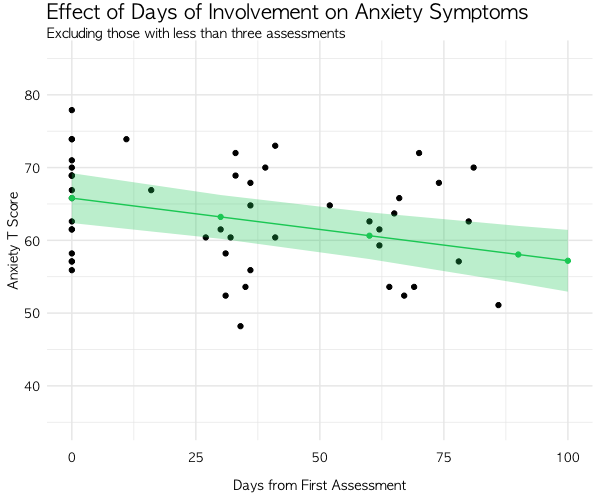


Figure S1. Linear mixed-effects model results demonstrating the main effect of days from first assessment on anxiety T-scores for those with three or more assessments.

| Table S2. Results of linear mixed-effects model for depressive symptom group with three or more assessments | | | |
| --- | --- | --- | --- |
|  | Depression T-score | | |
| *Predictors* | *Estimates* | *CI* | *P* |
| (Intercept) | 70.66 | 61.13 - 80.18 | **<.001** |
| Days from baseline | -0.04 | -0.25 - 0.17 | 0.665 |
| Random effects |  |  |  |
| σ^2^ | 51.30 |  |  |
| τ_00_ | 0.00 |  |  |
| N _Member ID_ | 3 |  |  |
| Observations | 9 |  |  |
| Marginal R^2^ / Conditional R^2^ | 0.026 / 0.026 | | |

*Note.* Statistically significant effects (*P* < .05) are bolded.


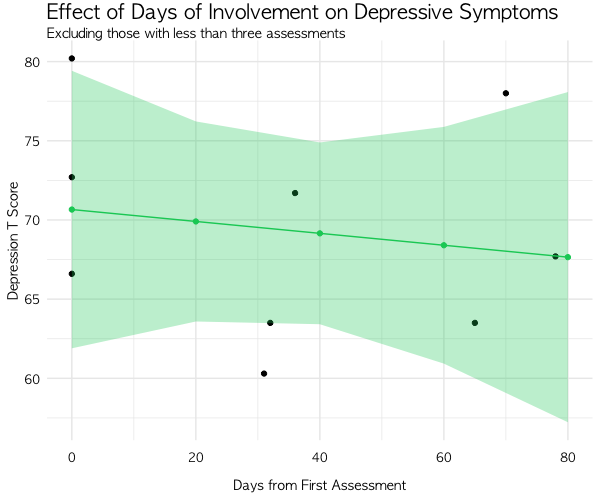


Figure S2. Linear mixed-effects model results demonstrating the main effect of days from first assessment on depression T-scores for those with three or more assessments.
